# The use of ion mobility mass spectrometry to probe modulation of the structure of p53 and of MDM2 by small molecule inhibitors

**DOI:** 10.3389/fmolb.2015.00039

**Published:** 2015-07-10

**Authors:** Eleanor R. Dickinson, Ewa Jurneczko, Judith Nicholson, Ted R. Hupp, Joanna Zawacka-Pankau, Galina Selivanova, Perdita E. Barran

**Affiliations:** ^1^The Michael Barber Centre for Collaborative Mass Spectrometry, Manchester Institute of Biotechnology, School of Chemistry, University of ManchesterManchester, UK; ^2^School of Chemistry, University of EdinburghEdinburgh, UK; ^3^Institute of Genetics and Molecular Medicine, CRUK Cancer Research Centre, University of EdinburghEdinburgh UK; ^4^Department of Microbiology, Tumor and Cell Biology, Karolinska InstitutetStockholm, Sweden

**Keywords:** conformational dynamics, ion mobility mass spectrometry, p53, MDM2, small molecule modulation

## Abstract

Developing drug-like molecules to inhibit the interactions formed by disordered proteins is desirable due to the high correlation of disorder with protein implicated in disease, but is challenging due in part to the lack of atomistically resolved and resolvable structures from conformationally dynamic systems. Ion mobility mass spectrometry (IM-MS) is well-positioned to assess protein ligand interactions along with the effect of a given inhibitor on conformation. Here we demonstrate the use of IM-MS to characterize the effect of two inhibitors RITA and Nutlin-3 on their respective binding partners: p53 and MDM2. RITA binds N-terminal transactivation domain of p53 (Np53) weakly, preventing direct observation of the complex in the gas phase. Nonetheless, upon incubation with RITA, we observe an alteration in the charge state distribution and in the conformational distributions adopted by Np53 in the gas phase. This finding supports the hypothesis that RITAs mode of action proceeds *via* a conformational change in p53. Circular dichroism corroborates our gas phase findings, showing a slight increase in secondary structure content on ligand incubation, and HDX-MS experiments also highlight the dynamic properties of this protein. Using the same approach we present data to show the effect of Nutlin-3 binding to the N-terminal domain of MDM2 (N-MDM2), N-MDM2 presents as at least two conformational families in the absence of Nutlin-3. Upon Nutlin-3 binding, the protein undergoes a compaction event similar to that exhibited by RITA on Np53. This multi-technique approach highlights the inherent disorder in these systems; and in particular exemplifies the power of IM-MS as a technique to study transient interactions between small molecule inhibitors and intrinsically disordered proteins.

## Introduction

The transcription factor p53, dubbed the Death Star (Vousden, 2000), is a multi-domain, intrinsically disordered protein (IDP) (Bell et al., 2002; Dawson et al., 2003). The protein comprises the disordered N-terminal domain (Np53) (Joerger and Fersht, 2008) containing the transactivation domain (residues 1–61) and the proline-rich domain (residues 62–94), the central DNA binding domain (residues 94–292), the tetramerization domain (residues 325–355) and the C-terminal regulatory domain (residues 363–393). It is strongly implicated in tumor suppression pathways, where it functions to block tumor development by triggering cellular senescence or apoptosis upon signals indicating DNA damage, oncogene activation, or telomere erosion (Vousden and Prives, 2009). Under non-stressed conditions, low p53 levels are tightly maintained by MDM2 (murine double minute 2). MDM2 is a ~55 KDa IDP with roles as an Ubiquitin E3 ligase, as a molecular chaperone and also in translational control. MDM2 comprises the disordered “lid” mini-domain (residues 1–24), (Uhrinova et al., 2005) the N-terminal domain (residues 25–109), the disordered central acidic domain (residues 221–276), the zinc finger domain (residues 299–331), and the C-terminal RING (really interesting new gene) domain (residues 430–480). MDM2 down regulates p53 activity in a negative autoregulatory feedback loop via three mechanisms; firstly, MDM2 blocks the transcription ability of p53 by direct binding through their respective N-terminal domains (Wu et al., 1993; Haupt et al., 1997). Secondly, MDM2 exports p53 from the nucleus and thirdly, targets p53 by Ubiquitination for degradation via the proteasome (Freedman and Levine, 1998; Tao and Levine, 1999). p53 N-terminal domain binds into the MDM2 N-terminal domain hydrophobic pocket as an amphipathic helix, with residues Ph19, Trp23, and Leu26 comprising a triad of required contacts which insert into the MDM2 binding cleft (Kussie et al., 1996).

Alteration of the p53 pathway is an almost universal hallmark of human cancers, with 22 million cancer patients living with abrogation of the p53 pathway, half of which display suppressed p53 function (Brown et al., 2009) and half of which exhibit p53 mutations. Cellular overexpression of MDM2 effectively abolishes p53 function, allowing unregulated cell cycle events in tumor cells. Inhibition of the p53:MDM2 complex is therefore a highly desirable therapeutic strategy; releasing, reactivating and stabilizing p53 levels, thus providing an attractive cancer therapy drug target. To date, numerous p53:MDM2 protein–protein interaction (PPI) antagonists have been identified, including cis-imidazolines (Vassilev et al., 2004; Vu et al., 2013), “stapled” peptides (Brown et al., 2012; Chang et al., 2013), terphenyls (Yin et al., 2005), oligobenzamides (Lu et al., 2006), spiro-oxindoles (Ding et al., 2006), chromenotriazolopyrimidine (Rew et al., 2012), Benzodiazepinedione (Grasberger et al., 2005), and Chromenotriazolopyrimidines (Allen et al., 2009). The cis-imidazoline Nutlin-3 is composed of enantiomers a and b, of which enantiomer a is 150 times more potent, and binds MDM2 in the p53 peptide groove, mimicking the three p53 residues responsible for the bulk of binding interactions (Vassilev et al., 2004). Nutlin-3 is effective in numerous cell lines, and is able to arrest or induce apoptosis in proliferating cancer cells with micromolar concentrations (Tovar et al., 2006). The drug candidate RITA (reactivation of p53 and induction of tumor cell apoptosis, NSC 652287) has been shown to restore wild-type p53 function in tumor cells by preventing the p53:MDM2 interaction (Issaeva et al., 2004). In contrast to the Nutlins, which bind MDM2 in its N-terminal hydrophobic pocket (Vassilev et al., 2004), RITA binds to p53 N-terminal domain with estimated *K*_D_ = 1.5 nM. It is hypothesized that RITA binds outside of the p53/MDM2 binding cleft, allosterically exerting its effect via a conformational change in the highly disordered N-terminus of p53 (Np53) (Issaeva et al., 2004).

Since its advent in the 1970's (Hogg and Kebarle, 1965; Kebarle and Hogg, 1965), the hybrid gas phase technique Ion Mobility-Mass Spectrometry (IM-MS) has gained credibility as a tool to study the conformations adopted by proteins and peptides in the gas phase. IM-MS is especially effective in its use for studying IDPs (Bernstein et al., 2004; Harvey et al., 2012; Pagel et al., 2013) due to its ability to observe conformations adopted by analytes on a millisecond time scale (Wyttenbach et al., 2001; McCullough et al., 2008). IM-MS provides information regarding charge, mass and shape of an analyte. The simplest setup of IM-MS is that of drift time IM-MS (DT IM-MS) (McAfee and Edelson, 1963). Ions are separated by their mobility (*K*) as they traverse a drift cell of known length filled with buffer gas to a known pressure and temperature. Ions travel down a weak electric field (5–50 V cm^−1^) colliding with buffer gas molecules which counter their progress until an equilibrium drift velocity, proportional to the electric field, is reached. The mobility (*K*) of an ion is the ratio between the drift velocity (*v*_d_) and the applied electric field (*E*). The mobility of an ion can be used to calculate the rotationally averaged collision cross section (CCS, Ω, Å^2^) using Equation (1) (Mason and McDaniel, 2005):

(1) $$K_{0}\;=\;\frac{3ze}{16N}{\left(\frac{2\pi }{\mu k_{B}T}\right)}^{0.5}\frac{1}{\Omega }$$

Where *K*_0_ is the reduced mobility; *z* is the ion charge state; *e* is the elementary charge; *N* is the gas number density; μ is the reduced mass of the ion-neutral pair; *k_B_* is the Boltzmann constant, and *T* is the gas temperature.

Here we employ native mass spectrometry, DT IM-MS, circular dichroism (CD) and hydrogen-deuterium exchange coupled to mass spectrometry (HDX-MS) to observe the conformations of N-terminal p53 domain (Np53) and the N-terminal domain of MDM2 (N-MDM2) both in the gas phase and in solution. We also probe the binding and conformational changes conferred by small molecule inhibitors; Nutlin-3 for N-MDM2, and RITA for Np53. Further information about DT IM-MS, CD and HDX-MS methodology can be found in the Supporting Information.

## Materials and methods

Expression and purification of both Np53 (residues 1–100) (Szekely et al., 1993; Bakalkin et al., 1995) and N-MDM2 (residues 1–126) (Worrall et al., 2010) have been previously described. Before the analysis reported here, the protein samples were thawed and dialysed in 50 mM ammonium acetate using Bio-RAD micro bio-spin chromatography columns (Bio-Rad Laboratories, Inc.). Concentrations of purified proteins were measured by the Thermo Scientific NanoDrop Spectrophotometer ND 1000 (Thermo Scientific, USA). Small molecule RITA [2,5-bis(5-hydroxymethyl-2-thienyl) furan, NSC 652287] was reconstituted in 100% IPA and stored at −20°C. Before analysis, RITA was thawed and diluted to 100 μM and an IPA concentration of 5% using 50 mM ammonium acetate. Nutlin-3 was reconstituted in 100% DMSO and stored at −80°C. Before analysis, Nutlin-3 was thawed and diluted to 500 μM and a DMSO concentration of 1% using 50 mM ammonium acetate.

MS and IM-MS experiments were performed on Np53 and N-MDM2 from solutions buffered with ammonium acetate (pH 6.8). Np53 samples were incubated with 5% IPA for 30 min at 37°C to account for the solvent present in the RITA sample. N-MDM2 samples were incubated with 0.5% DMSO for 30 min at room temperature to account for the solvent present in the Nutlin-3 sample. Binding experiments were performed on Np53 with RITA in a 1:2 protein:ligand ratio, samples were incubated for 30 min at 37°C. Binding experiments were performed on N-MDM2 and Nutlin-3 in a 1:10 protein:ligand ratio, samples were incubated for 30 min at room temperature. All MS and DT IM-MS data were acquired on an in-house modified quadropole time-of-flight mass spectrometer (Waters, Manchester, UK) (McCullough et al., 2008) containing a copper drift cell of length 5.1 cm. Ions were produced by positive nano-electrospray ionization (nESI) with a spray voltage of 1.3–1.62 kV. Helium was used as the buffer gas, its pressure measured using a baratron (MKS Instruments, UK). Buffer gas temperature and pressure readings (294.31–303.69 K and 3.518–3.898 Torr, respectively) were taken at each drift voltage and used in the analysis of drift time measurements. The drift voltage across the cell was varied by decreasing the cell body potential from 60 to 15 V, with arrival time measurements taken at a minimum of five distinct voltages. Instrument parameters were kept as constant as possible and are as follows: cone voltage: 114–119 V, source temperature: 80°C.

nESI tips were prepared in-house using a micropipette puller (Fleming/Brown model P-97, Sutter Instruments Co., USA) using 4″ 1.2 mm thin wall glass capillaries (World Precision Instruments, Inc., USA) and filled with 10–20 μL of sample.

Data was analyzed using MassLynx v4.1 software (Waters, Manchester, UK), Origin v9.0 (OriginLab Corporation, USA) and Microsoft Excel. Experiments were carried out in triplicate. Ion arrival time distributions were recorded by synchronization of the release of ions into the drift cell with mass spectral acquisition. The collision cross section distributions (CCSD) are derived from arrival time data using Equation (2) (Mason and McDaniel, 2005):

(2) $$\Omega_{\text{avg}}=\frac{{\left(18\pi \right)}^{1/2}}{16}{\left[\frac{1}{m_{b}}+\;\frac{1}{m}\right]}^{1/2}\frac{ze}{{\left(K_{B}T\right)}^{1/2}}\frac{1}{\rho }\frac{t_{d}V}{L^{2}}$$

Where Ω is the collision cross section (Å^2^); *m* and *m_b_* are the masses of the ion and buffer gas, respectively; *z* is the ion charge state; *e* is the elementary charge; *k_B_* is the Boltzmann constant; *T* is the gas temperature; ρ is the buffer gas density; *L* is the drift tube length; *V* is the voltage across the drift tube; and *t_d_* is the drift time. For these experiments where the CCS has been evaluated experimentally with helium as the buffer gas and using a drift tube with a linear field we use the convention ^DT^CCS_He_ to report our collision cross section values in the context of the mobility technique employed as well as the buffer gas used.

HDX-MS experiments were carried out using a fully automated LEAP autosampler system (HTS PAL, Leap Technologies, Carrboro, NC, USA) previously described (Chalmers et al., 2006; Zhang et al., 2009) and an online Acquity UPLC M-class HDX System (Waters Inc., Manchester, UK). Np53 and RITA were mixed at a 1:2 protein:ligand ratio and incubated for 30 min at 37°C before analysis. Stock protein solutions (50 μM Np53 ± 100 μM RITA, with 5% IPA) were diluted to 10 μM with equilibration buffer. 3.8 μl protein solution was incubated with D_2_O (54.2 μl labeling buffer) and incubated at 18°C for 15, 30, 60, or 120 s. Following deuterium on-exchange, 50 μl of the labeled protein solution was quenched by adding 50 μl of quench buffer at 1°C, and samples were passed across an immobilized pepsin column (enzymate BEH pepsin column, Waters Inc., Manchester, UK) at 100 μL min^−1^ (H_2_O + 0.1% formic acid, 20°C). The resulting peptides were trapped on a UPLC BEH C_18_ Van-Guard Pre-column (Waters Inc., Manchester, UK) and then gradient eluted (1 min loading time, 8–85% ACN + 0.1% formic acid gradient, 40 μl min^−1^, 1°C) across a UPLC BEH C_18_ column (Waters Inc., Manchester, UK) before undergoing electrospray ionization and analysis using a Synapt G2Si mass spectrometer (Waters Inc., Manchester, UK). Data was analyzed using ProteinLynx Global Server (PLGS) (Waters, Manchester, UK), Dynamx v1.0 (Waters, Manchester, UK) and Origin v9.0 (OriginLab Corporation, USA).

Hundred percentage of sequence coverage was obtained for Np53 ± RITA. Selected peptides were restricted to be present in all three repeats of 0 s incubation time experiments.

## Results

### Modulation of N-terminal p53 by RITA

**Figure 1:**
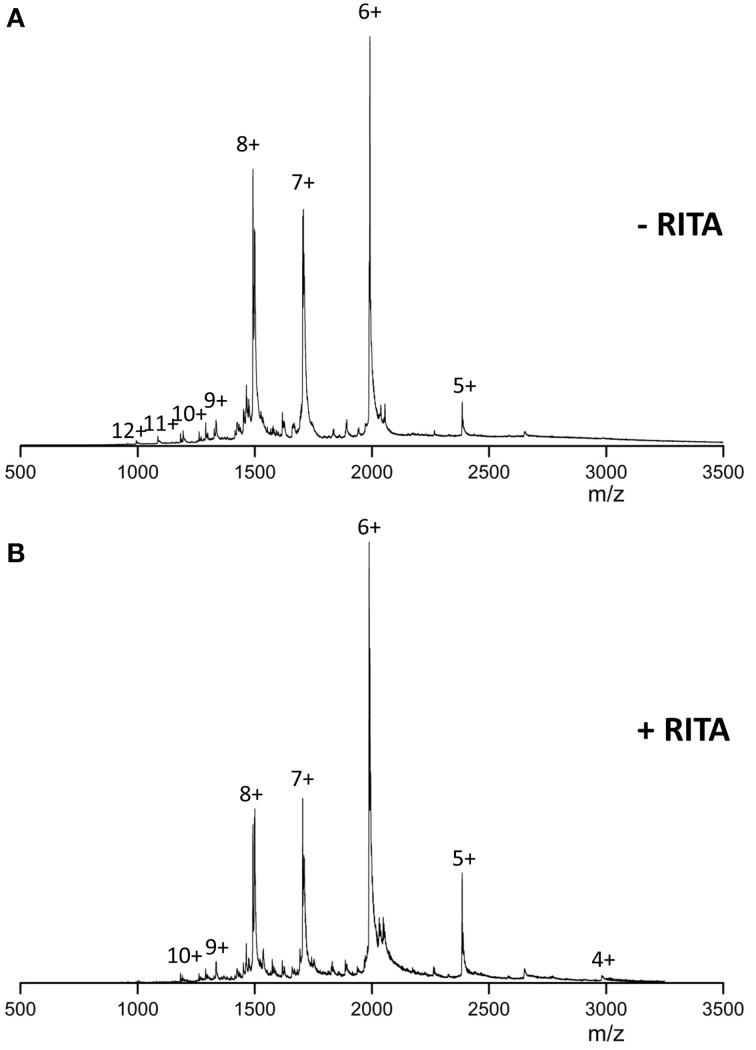
**n-ESI mass spectra recorded for (A) wild-type Np53 and (B) wild-type Np53 in the presence of RITA**. Np53 was incubated for 30 min at 37°C with addition of 5% IPA. Binding studies were carried out at a 1:2 Np53:RITA ratio, incubated for 30 min at 37°C with 5% IPA content.

In the absence of RITA and under near neutral solution pH conditions, the mass spectra of Np53 (Figure 1A) presents a broad monomeric charge state distribution (CSD) range 5 ≤ *z* ≤ 12, with three major signals corresponding to the ions [M+6H]^6+^, [M+7H]^7+^, and [M+8H]^8+^, of which the [M+6H]^6+^ species is most intense. Upon incubation of Np53 with RITA we observe a shift in the CSD toward lower charge states. Specifically, Np53 in the presence of RITA (Figure 1B) exhibits a significant decrease in intensity of the [M+7H]^7+^ and [M+8H]^8+^ species, along with an increase in the intensity of the [M+5H]^5+^ species, an appearance of the [M+4H]^4+^ species and a loss of the high charge states *z* >10. Although source conditions were carefully controlled to give gentle ionization of the sample, the Np53:RITA complex was not strong enough to be retained during desolvation at any protein:ligand ratio (data not shown).

**Figure 2:**
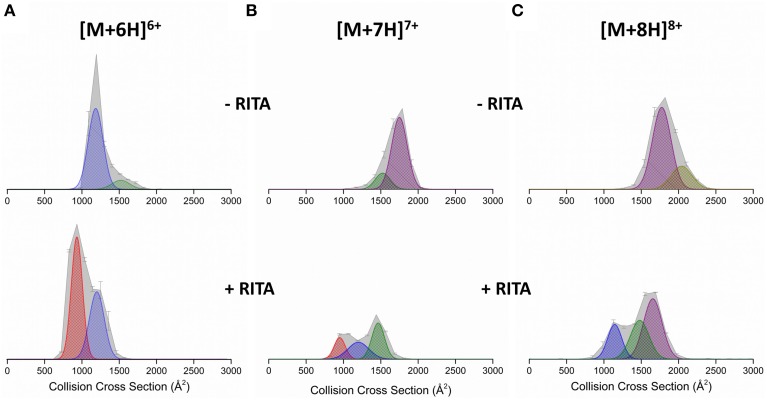
**Collision cross section distributions (^DT^CCSD_He_) arising from arrival time distributions (ATDs) at drift voltage 35 V for Np53 in the absence (top panels) and presence (bottom panels) of RITA**. Distributions shown for the **(A)** [M+6H]^6+^ **(B)** [M+7H]^7+^, and **(C)** [M+8H]^8+^ species. In the absence of RITA, Np53 was incubated with 5% IPA for 30 min at 37°C. Binding studies were carried out at a 1:2 Np53:RITA ratio incubated at 37°C for 30 min with 5% IPA. ^DT^CCSD_He_ are normalized to the intensity of the ion peak in the corresponding mass spectrum. Conformational families are denoted by hatched Gaussian curves showing novel compact conformational family C_0_ in red, compact conformational family C_1_ in blue, extended conformational family X in green, and unfolded conformational family U in purple.

DT IM-MS was performed on Np53 both in the absence and presence of RITA. The collision cross section distribution (^DT^CCSD_He_) (Figure 2A top panel) shows the Np53 [M+6H]^6+^ charge state presents as two conformational families; a more populated compact form (denoted C_1_, blue Gaussian distribution) centered at ~1250 Å^2^ and a low intensity extended form (denoted X, green Gaussian distribution) centered at ~1500 Å^2^. Two conformations are also observed for [M+7H]^7+^ (Figure 2B), which are assigned to X and a more intense larger distribution, centered at ~1750 Å^2^, which is assigned to an unfolded form of the protein (U, purple Gaussian distribution). [M+8H]^8+^ (Figure 2C), is also made up of U, along with low intensity signal from a still more extended form (U_2_, gold Gaussian distribution), although this latter distribution is poorly resolved. [M+9H]^9+^ (Figure S2) presents in three conformational families; X, U, and U2, of which the most extended U2 is most populated.

Upon incubation with RITA the ^DT^CCSD_He_ for [M+6H]^6+^ is significantly altered (Figure 2A bottom panel); we no longer observe the extended conformer X, observe a reduction in the population of compact conformer C_1_, and the induction of a highly populated novel conformational family centered at ~950 Å^2^, C_0_ (red Gaussian distribution). The [M+7H]^7+^ CCSD (Figure 2B) is also altered by the presence of RITA, with loss of conformer U, and induction of both conformers C_1_ and C_0_. This change is accompanied by a decrease in intensity of this charge state. [M+8H]^8+^ (Figure 2C) behaves similarly to [M+7H]^7+^, with loss of conformer U_2_, and induction of conformers X and C_1_. We observe an increase in the intensity of the [M+5H]^5+^ species (Figure S1) along with the appearance of a highly compact form of the protein C_0_. This compaction is evident in all charge states, for example [M+9H]^9+^ (Figure S2) has lost the population of the unfolded conformer U_2_ upon incubation with RITA, alongside a reduction in intensity of conformers X and U and induction of highly populated C_1_ conformational family. This alteration of the conformational spread as shown by the ^DT^CCSD_He_ is supported in solution by CD. Figure S3 (Supporting Information) shows the secondary structure content of Np53 increases upon incubation with RITA, supporting the hypothesis that RITA induces a novel conformer of Np53. Structural analysis using DiChroWeb (Lobley et al., 2002) using CONTILL algorithm (Provencher and Gloeckner, 1981; Sreerama and Woody, 2000) predicted that Np53 is 32% disordered, and upon incubation with RITA the level of disorder reduced to 28%.

**Figure 3:**
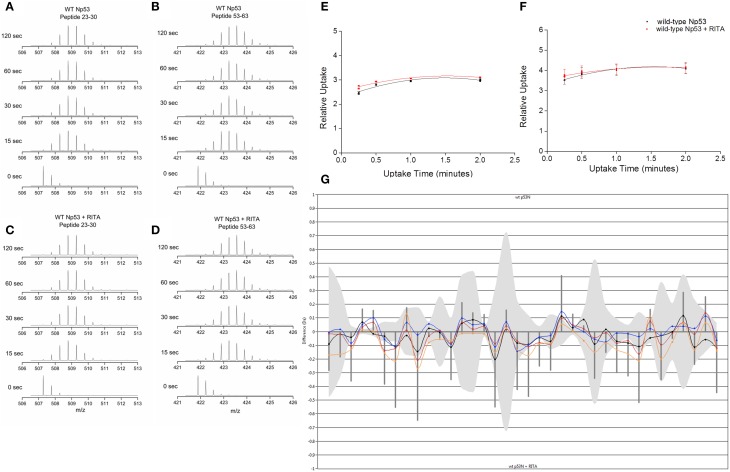
**(A,C)** Deuterium uptake mass spectra for Np53 peptide residues 23–30 at time points 0, 15, 30, 60, and 120 s in the absence **(A)** and presence **(C)** of RITA. **(B,D)** Deuterium uptake charts for Np53 peptide residues 53–63 at time points 0, 15, 30, 60, and 120 s in the absence **(B)** and presence **(D)** of RITA. **(E,F)** Relative deuterium uptake plots for Np53 in the absence (black points) and presence (red points) of RITA for peptide residues 23–30 and 53–63, respectively. **(G)** Butterfly plot of uptake difference comparison for Np53 in the absence and presence of RITA. X-axis denotes each peptide detected, in ascending residue number. Y-axis denotes uptake difference (Daltons). Colored lines show uptake difference at 15 (yellow), 30 (red), 60 (blue), and 120 (black) s incubation time. Gray band indicates the error associated with each peptide. Vertical lines are the summed difference of all time points for each peptide.

Hydrogen–deuterium exchange coupled to mass spectrometry (HDX-MS) was used to ascertain if the conformational changes induced by RITA could be mapped in the solution phase. Np53 was incubated for varying time points in deuterated buffer, and the mass shift of peptides was determined. Np53 shows a significant uptake of deuterium at the shortest experimental time point of 15 s for a large proportion of peptides detected (Figures 3A–D). From the mas spectrometry data of each peptide, we observe no significant difference between deuterium uptake in the absence or presence of RITA, as shown by the deuterium uptake curves for selected representative peptides residues 23–30 and 53–63 (Figures 3E,F, respectively). This indicates that we cannot sample the interconverting solution conformations for this highly dynamic protein over the longer timescale of the HDX-MS experiment. The butterfly plot in Figure 3G depicts the overall deuterium uptake differences between Np53 in the absence and presence of RITA. Each set of points along the x-axis represent a peptide, with time points denoted by different colored points and lines [15 (yellow), 30 (red), 60 (blue), and 120 (black) s incubation time]. Gray bands indicate the error in the uptake level and vertical lines indicate the sum of uptake differences for each time point. Several peptides show deuterium uptake differences slightly above the error, but all at <1 Da, indicating that this protein is highly dynamic with or without RITA, for example, the greatest deuterium uptake difference in Np53 in the absence and presence of RITA being 0.271 Da, for a peptide spanning residues 23–39 with a [M+2H]^2+^ charge state.

### Modulation of N-terminal MDM2 by Nutlin-3

**Figure 4:**
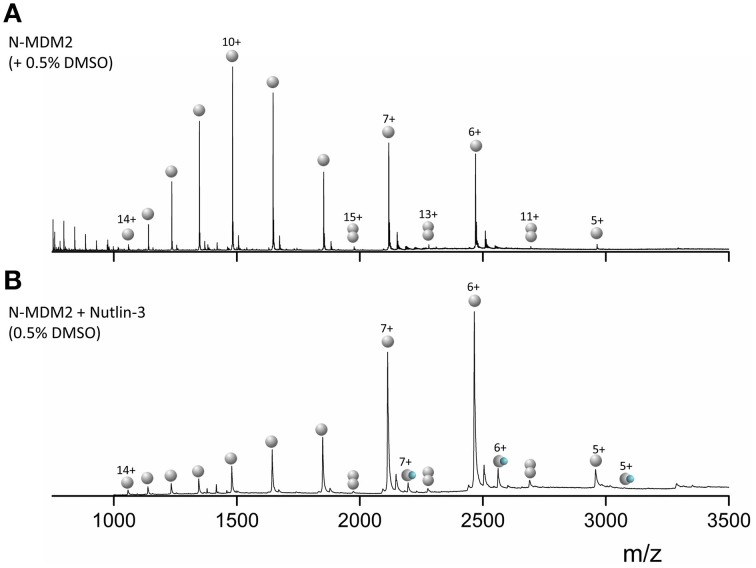
**nESI mass spectra of (A) 50 μM N-terminal MDM2 (residues 1–126) sprayed from 50 mM ammonium acetate + 0.5% DMSO. (B)** 50 μM N-MDM2 + Nutlin-3 in a 1:10 protein:ligand ratio, incubated for 30 min at room temperature. Final DMSO concentration 0.5%. Monomeric species are denoted by single gray spheres, dimeric species by two gray spheres and N-MDM2 bound to Nutlin-3 by a single gray sphere and small blue sphere.

Mass Spectra for MDM2 (Figure 4A) sprayed from native conditions with 50 mM ammonium acetate and 0.5% DMSO show a broad bimodal CSD spanning charge states 5 ≤ *z* ≤ 14. The most intense species is [M+10H]^10+^ with significant intensity also in [M+7H]^7+^ and [M+6H]^6+^. We observe low intensity [D+11H]^11+^, [D+13H]^13+^, and [D+15H]^15+^ dimers, which means that the species attributed to [M+5H]^5+^ will also contain some [D+10H]^10+^ (and the [M+6H]^6+^ some [D+12H]^12+^ etc.) but since the flanking unique m/z dimers are of an intensity of <5% we ignore this contribution. Upon incubation with Nutlin-3 (Figure 4B) we see a CSD shift toward the lower charge states, with the [M+6H]^6+^ species most intense, although the CSD range is retained. Binding of one Nutlin-3 molecule to MDM2 is observed at the [M+5H]^5+^, [M+6H]^6+^, and [M+7H]^7+^ charge states. The shift in the N-MDM2 CSD upon incubation with Nutlin-3 is substantially greater than that caused by DMSO alone (Figure S4, Supporting Information) suggesting that Nutlin-3 confers a structural or conformational change in N-MDM2.

**Figure 5:**
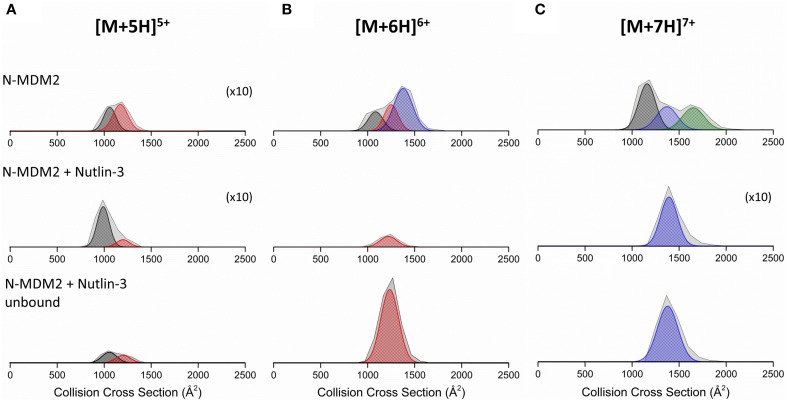
**Collision cross section distributions (^DT^CCSD_He_) derived from arrival time distributions (ATDs) for N-MDM2 sprayed from 50 mM ammonium acetate (+ 0.5% DMSO) for the (A) [M+5H]^5+^, (B) [M+6H]^6+^, and (C) [M+7H]^7+^ charge states**. Top panel represents N-MDM2, middle panel represents N-MDM2 bound to Nutlin-3 and bottom panel represents N-MDM2 incubated with Nutlin-3 but in apo-form with no small molecule bound. Proteins were incubated with Nutlin-3 in a 1:10 protein:ligand ratio, for 30 min at room temperature. CCSDs were taken at a drift voltage of 35 V and are normalized to the intensity of the ion species in the corresponding mass spectrum however to allow greater visibility, the [M+5H]^5+^, [M+5H]^5+^ bound to Nutlin-3 and [M+7H]^7+^ bound to Nutlin-3 ^DT^CCSD_He_ have been magnified X10. Conformational families are depicted by colored Gaussian curves; C_1_ (black), C_2_ (red), X (blue), and U (green).

DT IM-MS analysis reveals that N-MDM2 in the absence of Nutlin-3 presents as at least two conformational families at all charge states (Figure S5, Supporting Information). The [M+5H]^5+^ charge state (Figure 5A) presents as two conformers centered at ~1000 and ~1250 Å^2^, referred to as C_1_ (black Gaussian curve) and C_2_ (red Gaussian curve), respectively. The [M+6H]^6+^ charge state (Figure 5B) presents as three conformers, the compact C_1_ and C_2_ families and a more extended family, X (blue Gaussian curve) centered at ~1400 Å^2^. The [M+7H]^7+^ charge state (Figure 5C) exhibits conformational family C_1_ and X and also presents as a large conformer, U (green Gaussian curve) centered at ~1700 Å^2^. Upon binding to Nutlin-3, we see a change in the ^DT^CCSD_He_ of N-MDM2 for each charge state. The [M+5H]^5+^ charge state (Figure 5A, middle panel) shows retention of the compact conformer C_1_, but a significant decrease in the intensity of C_2_. The [M+6H]^6+^ charge state, when bound to Nutlin-3, undergoes a compaction event to produce a single conformational family centered at ~1250 Å^2^, corresponding to conformer C_2_ (Figure 5B, middle panel). This effect is again seen for the [M+7H]^7+^ charge state (Figure 5C, middle panel), which presents as a single conformer corresponding to conformer X when bound to Nutlin-3. These altered conformations remain, even when Nutlin-3 is not bound to N-MDM2. Figure 5 bottom panels show the ^DT^CCSD_He_ of N-MDM2 in the presence of Nutlin-3, but not bound in a complex. The [M+5H]^5+^ species undergoes a minor change in the ^DT^CCSD_He_ with an increase in conformational family C_2_ compared with the bound complex. Charge states [M+6H]^6+^ and [M+7H]^7+^ remain in the single conformational families C_2_ and X, respectively, even after the ligand is no longer bound.

## Discussion

The MS spectra for Np53 in the absence of RITA (Figure 1A) corroborates previous reports of disorder for the N-terminus of p53 (Bell et al., 2002; Dawson et al., 2003), a broad charge state range 5 ≤ *z* ≤ 12, indicative of a disordered system (Testa et al., 2011; Beveridge et al., 2014) with numerous residues available for protonation in solution. We are unable to preserve the binding of small molecule RITA to Np53, suggesting the binding is lower affinity than that reported previously (*K*_D_ = 1.5 nM, Issaeva et al., 2004) or that it proceeds principally by hydrophobic interactions that are significantly diminished in the absence of solvent, resulting in loss of ligand during desolvation. We observe a narrowing of the CSD for Np53 on ligand incubation, we also observe the isolated ligand (data not shown), which also supports our assertion of ligand dissociation during desolvation. This CSD shift toward lower charge states suggests conformational tightening induced by RITA, a hypothesis that is supported by DT IM-MS data. The ^DT^CCSD_He_ for Np53 is significantly altered in the presence of RITA at all charge states present, with loss of larger conformational families and induction of more compact conformers. We observe a compact conformer C_0_ for [M+5H]^5+^, [M+6H]^6+^, and [M+7H]^7+^ charge states, which is not present in the absence of RITA. Whilst the [M+8H]^8+^species does not contain any of the C_0_, it no longer contains conformer U, rather is populated by the more compact conformers C_1_ and X, although conformer X is poorly resolved. [M+9H]^9+^ undergoes loss of conformer U_2_ with induction of compact conformer C_1_ at a much lower ^DT^CCS_He_. The use of IM-MS to discern conformational tightening due to ligand binding has been previously reported, (Harvey et al., 2012) and along with these findings provides an exciting prospect as a method for screening inhibitors to conformationally dynamic systems. As RITA is predicted to bind outside of the p53:MDM2 hydrophobic binding pocket (Issaeva et al., 2004), it has been asserted that the observed inhibition proceeds *via* a conformational change, which in turn will allosterically prevent the binding of MDM2. Our IM-MS data is evidence for the conformational modulation of Np53 by RITA. The induction of a smaller conformation is corroborated by CD results, which show an increase in secondary structure, and a decrease in the disordered content when analyzed using the CONTILL algorithm. We do note, however, that the calculated differences in structural content predictions for Np53 are minimal, with only a 4% decrease in disordered content, this is less informative than the clear conformational change provided by IM-MS.

The use of HDX-MS reinforces the view that Np53 is conformationally dynamic in solution; high levels of deuterium uptake are observed after 15 s incubation, with very little further uptake at longer incubation times. This suggests that backbone amides are solvent exposed and free to exchange with deuterium. When uptake was compared after RITA incubation we observe no significant changes in deuterium uptake for Np53 (Figure 3G). While there are several peptides which exhibit deuterium uptake differences outside of the error, the greatest difference is 0.271 Da for a [M+2H]^2+^ peptide. As we see no difference greater than 1 Da, the mass difference between a hydrogen and deuterium atom, we can infer that there is no significant structural difference between Np53 in the absence and presence of RITA on the timescale of these experiments. Our shortest time step (15 s) is insufficient to observe the conformational changes occurring as the protein has enough time to rearrange back to its original conformations. In contrast, the isolated gas phase conformers exiting the electrosprayed droplets appear trapped in distinct conformers at least over the time scale of our IM-MS experiments. We estimate this time to be ~15 ms including the transmission of ions to our drift cell (McCullough et al., 2008), which is short enough to retain the conformational changes induced by RITA such that they can be observed. Both of the solution approaches *indicate* conformational flexibility and some slight change in structural content in the presence of the ligand, IM-MS provides a more definitive readout of the modulation of conformation to Np53 in the presence of RITA.

We can contrast the results observed for the RITA interaction with Np53 with that for the well-studied drug candidate Nutlin-3 with MDM2. N-MDM2 presents with a wide CSD (5 ≤ *z* ≤ 14), again suggesting a disordered protein. DT IM-MS shows that N-MDM2 presents in the gas-phase in at least two conformational families, potentially assignable to the previously reported “open” and “closed” position of the lid mini-domain (Uhrinova et al., 2005; Worrall et al., 2009, 2010). When incubated with Nutlin-3, we observe a substantial CSD shift toward the lower charge states which cannot be attributed to the effect of DMSO alone (Figure S4), again suggesting some conformational effect conferred by Nutlin-3 binding. We observe binding of Nutlin-3 to N-MDM2 over three charge states, [M+5H]^5+^, [M+6H]^6+^, and [M+7H]^7+^. As there is no binding to the more extended high charge states, Nutlin-3 may only be able to bind N-MDM2 in a compact conformation, which is transferred to the gas phase as low charge state complex. DT IM-MS analysis showed that Nutlin-3 configures N-MDM2 into a more compact and inflexible conformer. The [M+5H]^5+^ charge state retains both conformational families upon Nutlin-3 binding, however the larger conformer at ^DT^CCS_He_ ~1250 Å^2^ was greatly reduced. For the [M+6H]^6+^ and [M+7H]^7+^ ions, Nutlin-3 binding configures the protein into a single conformer with a narrow ^DT^CCSD_He_, indicating less dynamics. This single conformational family was centered at a ^DT^CCS_He_ ~1250 Å^2^ for [M+6H]^6+^, corresponding to conformational family C_2_, and ~1400 Å^2^ for [M+7H]^7+^ corresponding to family X. We see loss of both the C_1_ and X families for [M+6H]^6+^ and loss of C_2_ and U for [M+7H]^7+^, suggesting much lower flexibility of the protein when bound to Nutlin-3.

Interestingly, it appears as for Np53, that the ligand free N-MDM2 in the IM-MS experiments also retains a “memory” of its in solution Nutlin-3 bound state. ^DT^CCSD_He_ of N-MDM2, incubated with Nutlin-3 but in its apo-form, show similar conformers than those which retain binding of Nutlin-3 (Figure 5, bottom panels). This suggests that Nutlin-3 binds a higher proportion of analyte molecules than we observe, but is not retained fully during desolvation. The apo [M+5H]^5+^ species is not only compact, suggesting that it rearranges back to the free N-MDM2 conformer, or that some of it arises from a conformer in solution that is incapable of binding Nutlin-3. The apo [M+6H]^6+^ and [M+7H]^7+^ remain in tight, single conformational families, and a much lower proportion of the Nutlin-bound N-MDM2 presents in the [M+5H]^5+^ charge state (Figure 4) supporting our hypothesis that Nutlin-3 is unable to bind as well to the very compact conformer C_1_. For the larger conformational families, N-MDM2 seems unable to rearrange back to its original conformations within the timescale of desolvation and analysis.

## Conclusions

Multiple techniques have been used to probe the binding of small molecule inhibitors RITA and Nutlin-3 to N-terminal p53 (Np53) and N-terminal MDM2 (N-MDM2), respectively. Native mass spectrometry of Np53 shows a shift in the CSD toward the lower charge states and loss of the more extended charge states upon incubation with RITA. IM-MS of Np53 reveals two conformational families in the absence of RITA. Upon incubation with RITA, Np53 is configured into a novel, more compact conformer C_0_ with loss of the more extended conformational family. We are able to retain this conformational tightening in the gas-phase on the time scale of our DT IM-MS experiments, even though we are unable to preserve the RITA:Np53 complex in the gas phase. HDX-MS data highlights the disordered nature of Np53, with no discernible conformations visible on a longer timescale. Very little differences are noted between the deuterium on-exchange of Np53 in the absence and presence of RITA, and we are unable to locate RITA induced conformational changes.

The nESI mass spectrum of N-terminal MDM2 shows a wide range of charge states (5 ≤ *z* ≤ 14) indicative of a disordered protein (Testa et al., 2011; Beveridge et al., 2014). The bimodal distribution suggests the protein may possess a more compact and more extended conformer. Indeed, DT IM-MS results show the protein presents as at least two conformational families at all charge states. Upon incubation with Nutlin-3, we observe ligand binding to the forms of the protein that present to the gas phase with low charge states [M+5H]^5+^, [M+6H]^6+^, and [M+7H]^7+^, suggesting selective binding to a compact conformer of MDM2, or possibly that more extended forms lose Nutlin-3 on transfer to the gas phase. The bound species of MDM2 are compact at all three charge states, with [M+6H]^6+^ and [M+7H]^7+^ forming a single conformational family centered at a ^DT^CCS_He_ of the middle conformational family exhibited by apo-N-MDM2. These conformational changes are likely retained by ions which lose the bound Nutlin-3 molecule during desolvation, indicating that the protein is unable to rearrange during the experiment. IM-MS is presented as a promising technique able to track conformational changes in unstructured proteins on a millisecond timescale.

### Conflict of interest statement

The authors declare that the research was conducted in the absence of any commercial or financial relationships that could be construed as a potential conflict of interest.
