# Supplementary material for: The use of ion mobility mass spectrometry to probe modulation of the structure of p53 and of MDM2 by small molecule inhibitors
Source: Front Mol Biosci. 2015 Jul 10;2:39. doi: 10.3389/fmolb.2015.00039 (PMC4498441; doi:10.3389/fmolb.2015.00039)
Supplement: Supplementary file 1 [file DataSheet1.PDF]

Supplementary Material

The use of Ion Mobility Mass Spectrometry to probe Modulation of Function of p53 and of MDM2 by Small Molecule Inhibitors

E. R. Dickinson[1], Ewa Jurneczko[2], Judith Nicholson[2], T. R Hupp[3], J. Zawacka-Pankau[4], G. Selivanova[4] and P. E. Barran[1]

[1]The Michael Barber Centre for Collaborative Mass Spectrometry Manchester Institute of Biotechnology, University of Manchester, Manchester, M1 7DN, U.K.

[2]School of Chemistry, University of Edinburgh, Edinburgh, EH9 3JJ, U.K.

[3]Institute of Genetics and Molecular Medicine, CRUK Cancer Research Centre, University of Edinburgh, Edinburgh EH4 2XR, U.K.

[4]Department of Microbiology, Tumor and Cell Biology, Karolinska Institutet, Stockholm, Sweden

*** Correspondence:** Perdita Barran, The Michael Barber Centre for Collaborative Mass Spectrometry Manchester Institute of Biotechnology, University of Manchester, Manchester, M1 7DN, U.K. Perdita.barran@manchester.ac.uk

1. Supplementary Information

1.1. IM-MS Theory

Ion mobility coupled to mass spectrometry (DT IM-MS) was utilised to distinguish the conformational families present in Np53. IM-MS separates analytes on the basis of their mass (m), charge (z) and shape to give a rotationally averaged collision cross section (CCS, Ω, $Å^2$). Ions are separated by their mobility (k) as they pass through a drift cell filled with a buffer gas of known temperature and pressure. A weak electric field (E) (5-50 V cm^{-1}) provides the forward motion of the ions along the drift cell, whilst collisions with buffer gas molecules slow the progress until ions reach a constant drift velocity (υ_d), allowing calculation of the mobility using Equation 1:

$$\upsilon_d = KE \qquad (1)$$

The mobility of an ion is often expressed as reduced mobility (K_0) which is the measured mobility K standardised for temperature (T), 273 K and pressure (P), 760 Torr. Using this parameter, we can calculate the buffer gas dependent CCS using Equation (2).

$$K_0 = \frac{3ze}{16N}\left(\frac{2\pi}{\mu k_B T}\right)^{0.5}\frac{1}{\Omega} \tag{2}$$

Where K_0 is the reduced mobility, z is the ion charge state, e is the elementary charge, N is the gas number density, μ is the reduced mass of the ion-neutral pair, k_B is the Boltzmann constant, T is the gas temperature and Ω is collision cross section.

The raw arrival time (t_a) of an ion includes the time the ion spends outside the drift cell but within the mass spectrometer, also known as the dead time (t_0). This can be calculated by taking an average of the intercept of a linear plot of average arrival time versus pressure/temperature and the drift time can be calculated using Equation 3:

$$t_D = t_a - t_0 \tag{3}$$

The CCS of a protein conformer can be used in comparison to coordinates obtained from biophysical techniques such as X-ray crystallography or NMR and from co-ordinates generated from computational studies [1-3].

1.2. HDX-MS Theory

Hydrogen deuterium exchange coupled to mass spectrometry (HDX-MS) was used to monitor solution phase conformational changes in Np53 in the absence and presence of RITA. In a HDX-MS experiment, the target protein is incubated with excess D_2O labelling buffer and on-exchange of solvent deuterons occurs for a set length of time. The continuous on-exchange labelling is then followed by quenching with a low pH buffer. Subsequently, the target protein is digested using an acid stable protease such as pepsin. Desalting and separation of the peptides by reversed-phase LC is followed by analysis using electrospray ionisation (ESI) mass spectrometry. The quench and LC steps of the experiment are carried out at low temperature (~0 °C) to minimize the back-exchange of Deuterium to Hydrogen. The digestion is carried out at the optimum pepsin digestion temperature (~20 °C) but kept to a minimum of time. Analysis of the resulting peptides monitors the deuteration level as a function of time by calculation of the mass shift in the isotopic distributions of individual peptides, enabling production of uptake graphs. Exposure of a protein to a D_2O containing environment will allow both side-chain and backbone Hydrogens to exchange freely to Deuterium, increasing the mass of the protein by 1 Da. Regions of the protein which are protected from the D_2O solution, by ligand binding or the fold of the protein, exhibit lower levels or a slower rate of on-exchange compared with solvent accessible backbone amides, which exchange freely and quickly. This can be used to relate to conformational dynamics of the protein on the timescale of the experiment. Uptake rates can be mapped onto protein structures created from x-ray crystallography or NMR data points.

1.3. Materials and Methods

1.3.1. Protein expression and purification

The expression and purification of MDM2 1-126 has been described previously [4] The expression and purification of wild-type N-terminal p53 (Np53) purification has also been described previously [5, 6]. In brief both proteins were expressed as glutathione-*S*-transferase-tagged proteins and purified from soluble *Escherichia coli* lysates. Cells were lysed and the proteins cleaved off column. The GST tag was also cleaved from the protein and all samples were snap-frozen in liquid nitrogen and stored in 25 μL aliquots at -80 ºC prior to analysis described in the main text.

1.3.2. HDX-MS buffer and pH

The equilibrium buffer (pH 7) was made up as 5 mM di-potassium hydrogen phosphate and 5 mM potassium di-hydrogen phosphate prepared in H_2O.. The Labelling buffer (pH 6.66) used 5 mM di-potassium hydrogen phosphate and 5 mM potassium di-hydrogen phosphate prepared in D_2O. For the low pH quench buffer (pH 2.66) 50 mM di-potassium hydrogen phosphate and 50 mM potassium di-hydrogen phosphate were prepared in H_2O. The pH of all buffers were measured with a Jenway 3505 pH meter.

1.3.3. Circular dichroism

Proteins were prepared to 50 µM using 50 mM ammonium acetate with 5% IPA. Protein samples were incubated with RITA which was diluted with ammonium acetate to 100 µM and an IPA concentration of 5%. Np53 proteins in the presence and absence of RITA were incubated for 30 minutes at 37 °C prior to analysis.

Circular dichroism (CD) spectra were acquired using a Chirascan CD spectrometer (Applied Photophysics, Ltd.) in the far UV region 180-260 nm with a spectral bandwidth of 1 nm, 0.5 nm step size and 0.1 mm path length. Three repeats of all experiments were taken and an average used. Data was analysed using Chriascan v 4.2.17 (Applied photophysics Ltd), Pro Data viewer (Applied photophysics Ltd) and CDNN v2.1 (Gerald Böhm).

2. Supplementary Figures

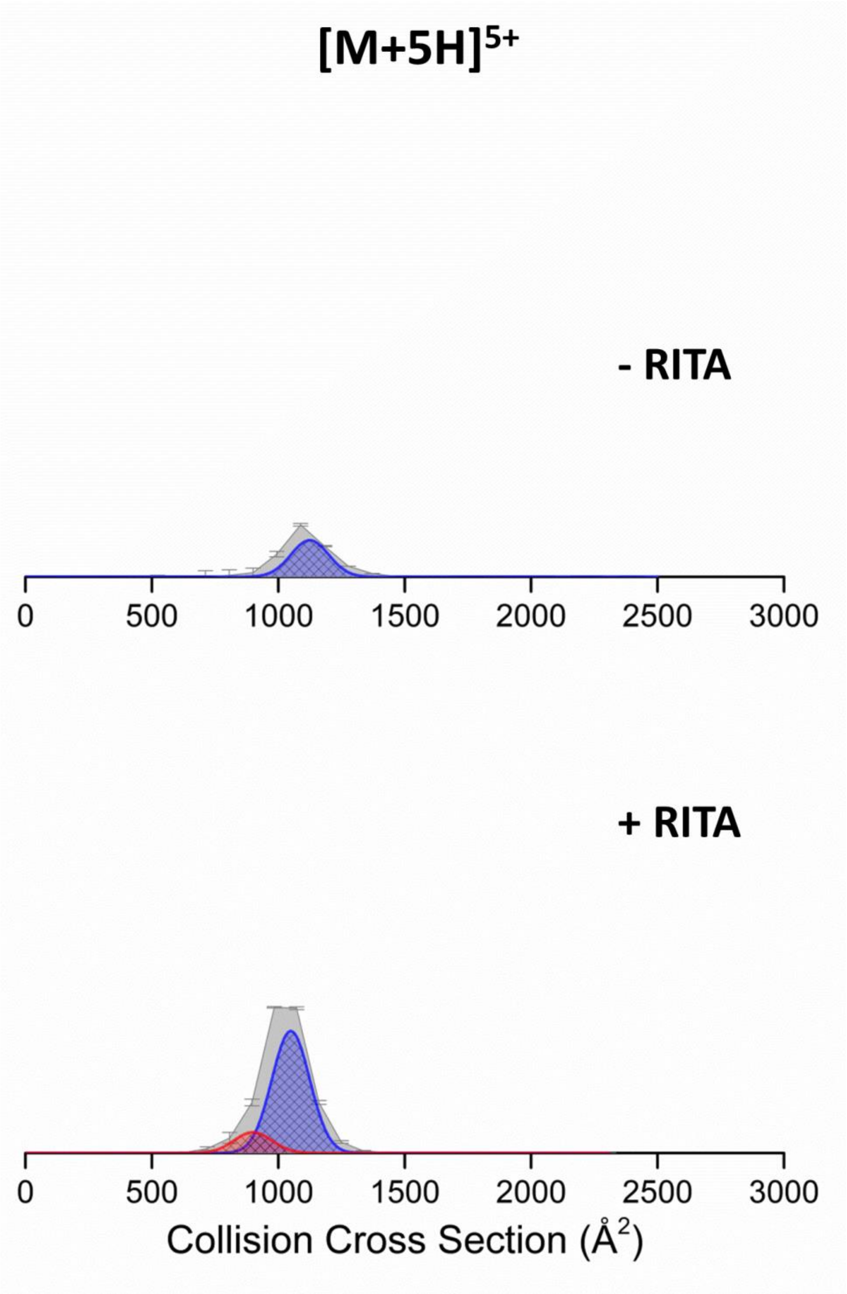

Figure S1. Collision cross section distribution (CCSD) derived from arrival time distributions (ATDs) for the $[M+5H]^{5+}$ charge state of wild-type Np53. Protein was analysed in the absence (top

panel) of RITA and in a 1:2 protein:ligand ratio with RITA (bottom panel). Both samples were incubated for 30 minutes at 37°C with 5% IPA. CCSDs were taken at a drift voltage of 35 V. Hatched Gaussian curves indicate conformational families C_0 (red) and C_1 (blue)CCSD intensity is normalized to the intensity of the ion peak in the mass spectrum.

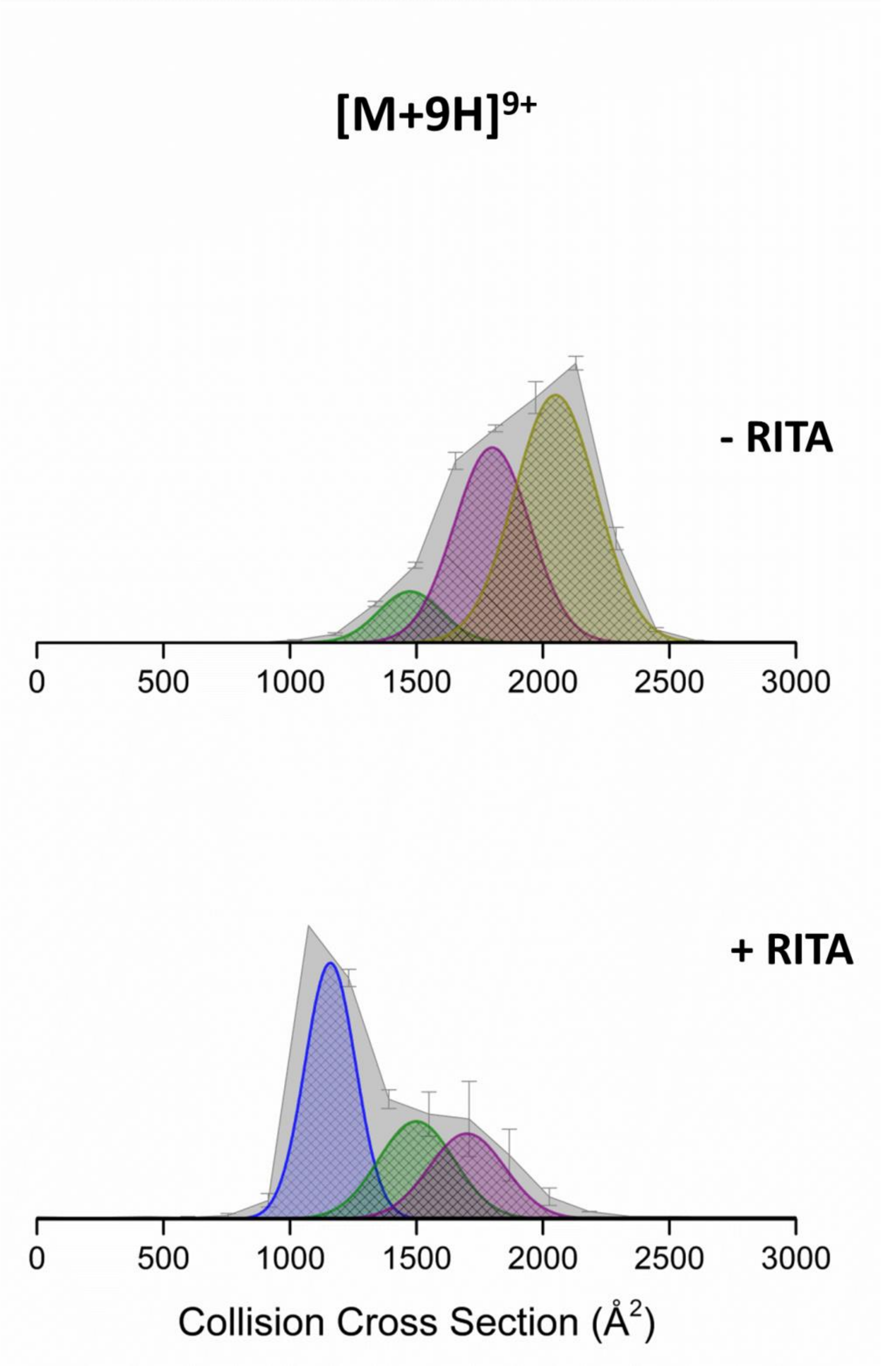

Figure S2. Collision cross section distribution (CCSD) derived from arrival time distributions (ATDs) for the $[M+9H]^{9+}$ charge state of wild-type Np53. Protein was analysed in the absence (top

panel) of RITA and in a 1:2 protein:ligand ratio with RITA (bottom panel). Both samples were incubated for 30 minutes at 37°C with 5% IPA. CCSDs were taken at a drift voltage of 35 V. Hatched Gaussian curves indicate conformational families present; C_1, X,U and U_2 in blue, green, purple and gold respectively. CCSD intensity is normalized to the intensity of the ion peak in the mass spectrum, however $[M+9H]^{9+}$ CCSD intensity is x10 to allow visibility of conformers present.

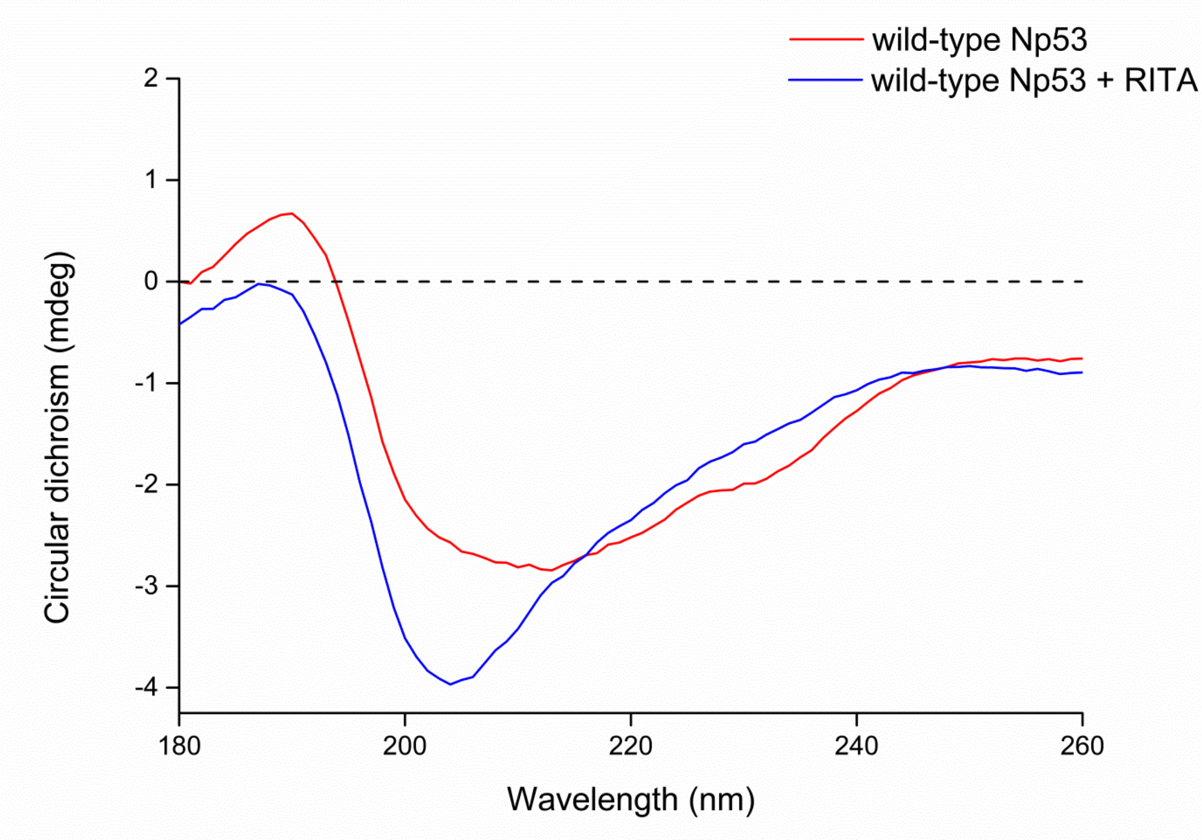

Figure S3. Circular dichroism spectra of wild-type Np53 (incubated at 37 °C for 30 minutes with 5% IPA) and wild-type Np53 incubated with RITA (1:2 protein:ligand incubated at 37 °C for 30 minutes) in red and blue, respectively.

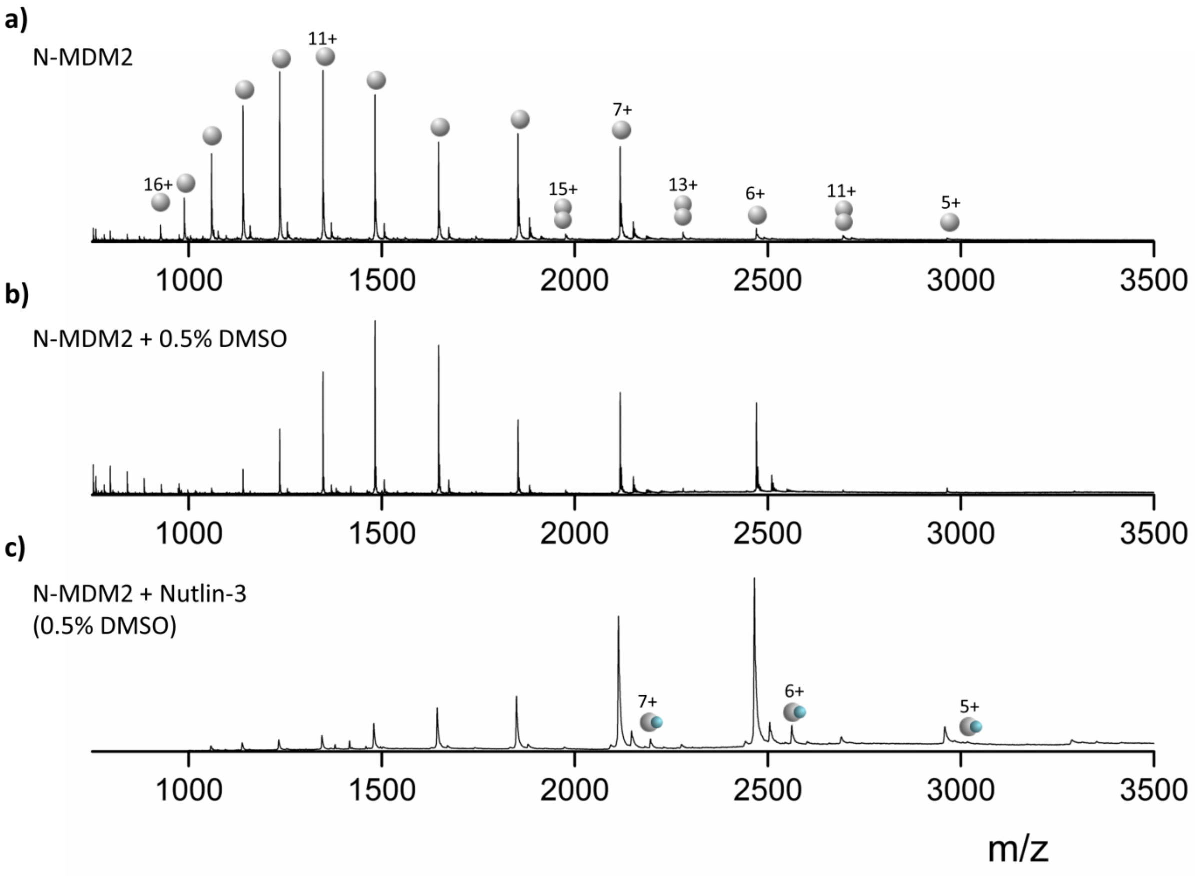

Figure S4. nESI mass spectra of a) 50 µM N-MDM2 in 50 mM ammonium acetate b)50 µM N-MDM2 + 0.5% DMSO and c) 50 µM N-MDM2 : 500 µM Nutlin-3 (0.5% DMSO). Single grey spheres denote monomeric species, double grey spheres denote dimeric species, and blue small spheres denote Nutlin-3 molecules.

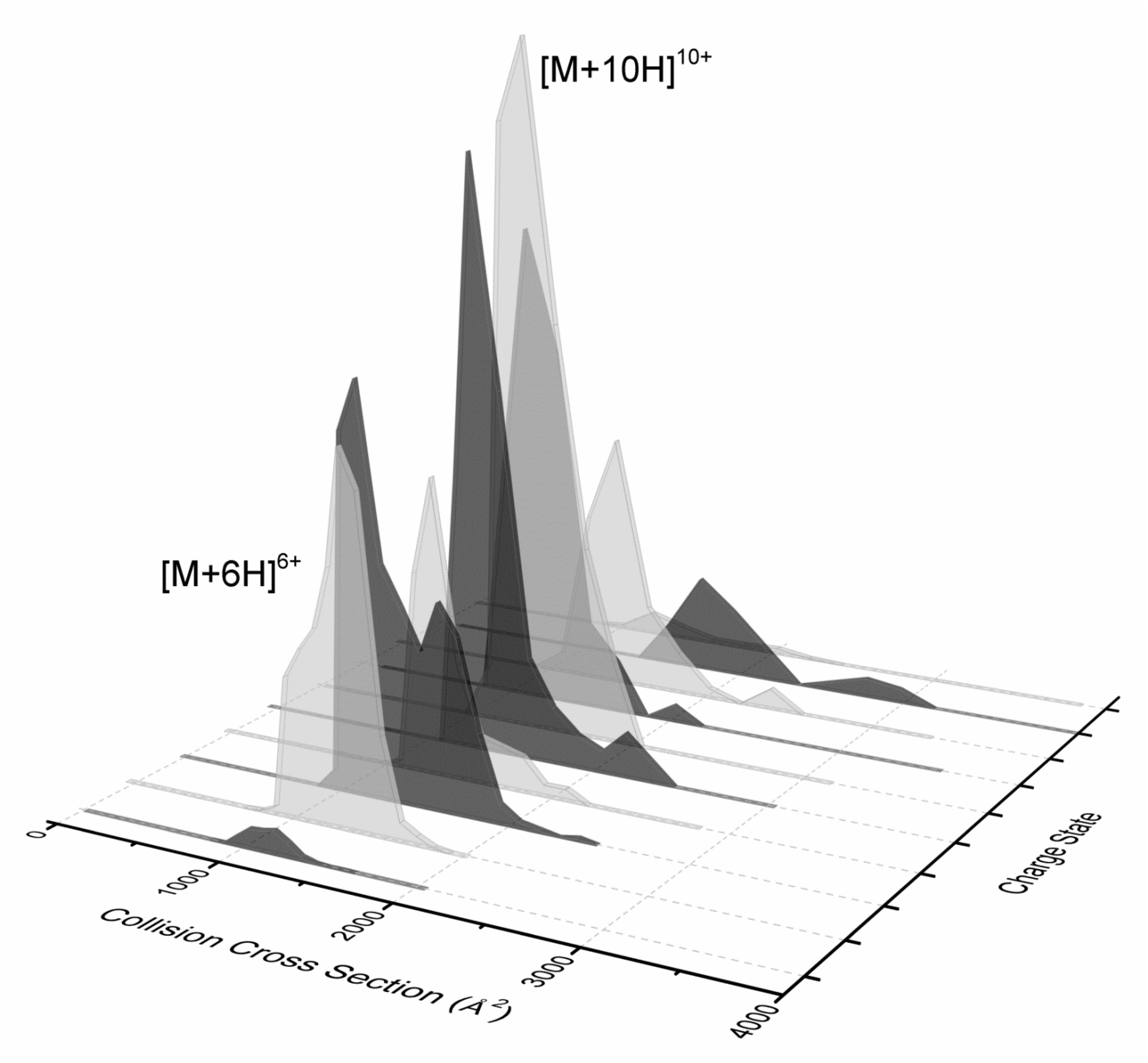

Figure S5. Waterfall plot representing in vacuo conformations of N-terminal MDM2 + 0.5% DMSO sprayed from 50 mM ammonium acetate. The x, y, z axis show the collision cross section ($^{DT}CCS_{He}$, Å^2), charge state (range: $5 \leq z \leq 14$) and the relative intensity, respectively. DT IM-MS data taken at a drift voltage of 35V is shown.

1. Harvey, S.R., et al., *Small-molecule inhibition of c-MYC:MAX leucine zipper formation is revealed by ion mobility mass spectrometry.* J Am Chem Soc, 2012. **134**(47): p. 19384-92.
2. Wu, C., et al., *The Structure of Aβ42 C-Terminal Fragments Probed by a Combined Experimental and Theoretical Study.* Journal of Molecular Biology, 2009. **387**(2): p. 492-501.
3. Knapman, T.W., et al., *Considerations in experimental and theoretical collision cross-section measurements of small molecules using travelling wave ion mobility spectrometry-mass spectrometry.* International Journal of Mass Spectrometry, 2010. **298**(1–3): p. 17-23.
4. Worrall, E.G., et al., *The effects of phosphomimetic lid mutation on the thermostability of the N-terminal domain of MDM2.* J Mol Biol, 2010. **398**(3): p. 414-28.
5. Bakalkin, G., et al., *p53 binds single-stranded DNA ends through the C-terminal domain and internal DNA segments via the middle domain.* Nucleic Acids Research, 1995. **23**(3): p. 362-369.
6. Szekely, L., et al., *EBNA-5, an Epstein-Barr virus-encoded nuclear antigen, binds to the retinoblastoma and p53 proteins.* Proceedings of the National Academy of Sciences, 1993. **90**(12): p. 5455-5459.
